# Real-time diagnosis of sentinel lymph nodes involved to breast cancer based on pH sensing through lipid synthesis of those cells

**DOI:** 10.1042/BSR20200970

**Published:** 2020-06-08

**Authors:** Zohreh S. Miripour, Parisa Aghaee, Fereshteh Abbasvandi, Parisa Hoseinpour, Mohammad Parniani, Mohammad Abdolahad

**Affiliations:** 1 Nano Electronic Center of Excellence, Cancer electronics Research center, Nano Bio Electronic Devices Lab., School of Electrical and Computer Engineering, Faculty of Engineering, University of Tehran,14395/515, Tehran, Iran; 2 Nano Electronic Center of Excellence, Thin Film and Nanoelectronics Lab, School of Electrical and Computer Engineering, College of Engineering, University of Tehran, P.O. Box 14395/515, Tehran, Iran; 3 ATMP Department, Breast Cancer Research Center, Motamed Cancer Institute, ACECR, P.O. Box 15179/64311, Tehran, Iran; 4 Central Lab of Breast Pathobiology, SEPAS Lab, Tehran, Iran

**Keywords:** bicarbonic acid, cancer, fatty acid oxidation, frozen pathology, metabolism-based sensor, pH sensing

## Abstract

Lipid synthesis is the recently found metabolism of cancer cells after their metastasis to lymph nodes (LNs). Carbonic acid is the main byproduct of the lipid metabolism in such cells which resulted in acidification of LN ambient. Hence, calibrated pH sensing could be a diagnostic method to find involved LNs. Here, we designed a simple pH sensing method by a syringe containing sterile PBS and embedded by litmus paper to intraoperatively check the pH of LN fluid. Injected phosphate buffer saline (PBS) would homogenize the LN fluid and litmus needle would detect the pH of the LN. We presented an experimental pathological calibration for the pH values in correlation with cancerous states of the LNs. This system named metabolism based metastatic lymph diagnoser (MMLD) could be a real-time noninvasive tool for precise and fast diagnosis of involved LNs.

## Introduction

Lymph nodes are small glands that filter lymphatic fluid (the clear fluid that circulates through the lymphatic system) [1]. The lymphatic system, part of the immune system, is a network of ducts that carry the lymphatic fluid (LF). LF also contains white blood cells called lymphocytes, fats, and proteins. The lymphatic system extracts LF from the body, and after purifying it from infectious organisms and abnormal cells carries it into the bloodstream [2]. In many types of cancers, especially breast carcinoma, the lymph node is the first location being invaded by cancer cells through lymphatic vessels [3]. Hence, breast tumor invasion to the sentinel lymph node (SLN) is the first sign of disease progression and metastasis. Detection of lymph node metastasis is crucial in the designation of treatment protocol by the oncologist [4]. Although the SLN involvement can be found during an interventional radiology (ultrasonography and biopsy) in many cases, it is mandatory to dissect the SLNs (either it was involved or free in biopsy result) in patients with invasive breast carcinoma to be sent for intraoperative frozen section [5,6]. If the SLN was involved, the surgeon must dissect at least six auxiliary LNs (ALNs) and if the SLNs were free from cancer cells it is not mandatory to dissect further lymph nodes [7–9]. So, we can prevent from removing normal ALNs or remaining involved ALNs which are crucial in programming the treating procedure of the patient [10,11].

Currently, the only common method for the detection of metastatic cancer cells in the lymph (sentinel and auxiliary) nodes during surgery is the frozen pathology which is highly dependent on the experience and skills of the pathologist. Due to the lack of time in the frozen section to respond to the surgeon, just part of the removed lymph node (due to color, stiffness, and firmness) is often selected for frozen examination [12]. So, in some cases, pathological mistakes in missing an involved LNs might be occurred in the frozen section (cancer cells might be missed and assumed as histiocytes). Based on these limitations, some new studies were introduced to detect the presence of metastatic cancer cells in lymph nodes.

One of these works is to inject an anti-epidermal growth factor (EGFR) or anti-herceptin2 (HER2) fluorescent substances followed by a 99mTc-phytate radioactive material around the mouse primary tumor [13,14]. Using microwave waves (with a bandwidth of up to 30 GHz) to detect cancerous cells in the lymph node was another reported method which took 15–30 min after wave ablation [15].

Similarly, tissue impedance imaging techniques were also used to detect cancer cells in the lymph nodes. The average time to scan an area by this method is approximately 5–10 min [16].

Although above methods made many improvements in intraoperative diagnosis of metastatic SLNs, still we haven’t a real-time noninvasive, simple, and reliable method to detect metastatic lymph nodes.

Here, we reported an accurate simple method to diagnose involved LNs based on pH sensing by a litmus paper (ColorpHast® pH Test Strips, Merck, Germany) embedded into a gauge 18 syringe needle (named as Metabolism based Metastatic Lymph Diagnoser (MMLD)), without any pre-preparation process which declared LN metastasis in less than 30 s. This process has been based on acidification of involved LNs due to fatty acid oxidation metabolism of cancer cells in LNs which was published recently [17]. By calibrating the live pH of SLNs by pathological calibration, we can diagnose the metastatic SLNs with no need for any lymph node dissection prior to diagnosis. Hence if in future it passed the required standards, we can maintain the free SLNs for the patient. Through frozen method even if the SLN was free from the tumor, the patient lost his/her SLNs which could cause many side effects for him/her such as lymphedema, lost or decreased sensation in the back of the arm or armpit region and tingling, numbness, stiffness in armpit and etc. [18–20].

## Materials and methods

### Fabrication of MMLD for *in vitro* assays

The system contains a micro-syringe (gauge 18, an inner diameter of the needle is 838 µm) filled by 100 µl of injectable sterile PBS and a syringe needle embedded by a thin litmus paper. The mechanism was entering the needle, injection of PBS rubbing the LNs, and checking the color of litmus paper which indicates the acidity or basicity of the lymph node fluid based on lipid synthesis of cancer cells in LNs. This test can be individually repeated by three needles and if even one of the needles showed acidic pH with pH lower than 6 (as experimentally was calibrated), then the LN would be declared as involved LNs.

### *In vitro* sample collection from the patients

Patients provided consent according to an ethically approved protocol (IR.TUMS.VCR.REC.1397.532) at our breast cancer central clinics and assistant hospitals. *In vitro* fresh lymph node samples prepared from 25 patients’ candidate for breast cancer were recorded from 2018-10-30 to 2019-11-30.

### Staining process using hematoxylin and eosin

Hematoxylin and eosin (H&E) staining is the most routine staining procedure in histopathology. This method is a combination of two dyes. H&E staining is used to demonstrate the nucleus and cytoplasmic inclusions in clinical specimens. Hematoxylin, which contains Alum, stains the nucleus in light blue. In the presence of an acid, the dye turns into the red. Therefore, by treating the tissue with an acid solution, the differentiation is achieved. In the bluing step, the initial soluble is converted to red color within the nucleus to an insoluble blue color. By utilizing Eosin, the counterstaining is done, which imparts pink color to the cytoplasm. The initial step of the H&E staining process is deparaffinized a tissue section and flaming the slide on a burner and placing it in the xylene. The process treatment must be repeated afterward, and the hydration process should be done. To hydrate the tissue section, one should be passing it through a decreasing concentration of alcohol baths and water (100%, 90%, 80%, and 70%). Then the sample should be stained in hematoxylin for approximately 3–5 min, in the next step, it should be washed in running tap water until sections ‘blue’ for 5 min or less. In the next level, the sample should be placed in 1% acid alcohol (1% HCl in 70% alcohol) for 5 min. Subsequently, the sample should be washed in running tap water until it turns into blue again by dipping in an alkaline solution (e.g., ammonia water) followed by tap water washing process. Furthermore, in 1% Eosin Y for 10 min, the sample is stained and should be washed in tap water for 1–5 min. Finally, to dehydrate the sample, one should dip it in increasing concentration of alcohol and clear in xylene.

## Result and discussion

The metabolism of cancer cells metastasize to LNs is very complicated. Recently, Lee et al. [17] used comparative methods of transcriptional analysis of mRNAs between breast primary tumors and those metastasized to lymph nodes in mouse models and found that cancer cells metastasized to LNs changed their metabolism from glycolysis to fatty acid oxidation (FAO). Expression of Yes-Associated Protein (YAP) selectively activates FAO-related genes in metastatic lymphatic tumors. The study of genes that are activated in tumors that have metastasized to the lymph will stimulate metabolism toward FAO rather than glycolysis [21].

Oxidation of fatty acids occurs in three stages. First, β-oxidation of fatty acids breaks down the two-carbon units (α and β carbons) of the carboxyl end of the fatty acyl-CoA and forms acetyl-CoA. This reaction continues until the entire fatty acyl chain gets broken down to acetyl-CoA molecules. Then, acetyl groups produced from β-fatty acid oxidation participate in the Krebs cycle and form NADH and FADH_2_. Moreover, the acetyl-CoA, formed from reactions, enters the Krebs cycle to oxidize carbon dioxide and water. Finally, the reduced NADH and FADH_2_ coenzymes are oxidized by giving protons and electrons to the oxygen present in the mitochondria to produce ATP by oxidative phosphorylation in the electron transport cycle [22–25].

**Figure 1:**
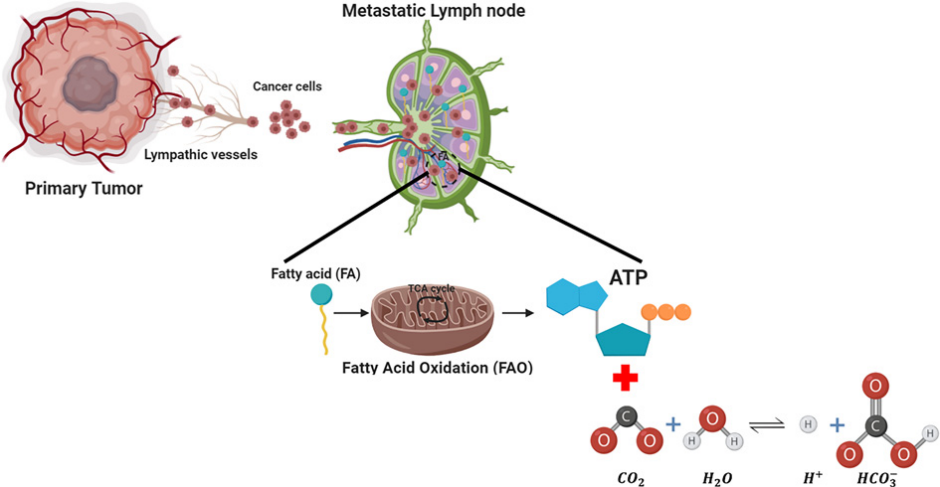
The schematic diagram for the mechanism of metastatic cancer cells to the lymph node Expression of YAP selectively activates FAO-related genes in lymphatic metastatic cancer cells and they used the plentiful fatty acids as fuel [17].

Also, released CO_2_ molecules during lipid synthesis of cancer cells would be dissolved in lymphatic fluids and resulted in the production of HCO_3_^−^ (bicarbonate) and H^+^ ions through below reversible reaction [26] (Figure 1).

$$CO_{2}+H_{2}O\Leftrightarrow H^{+}+HCO_{3}^{-}$$

Therefore, the environment of a metastasized lymph node would be acidic.

**Figure 2:**
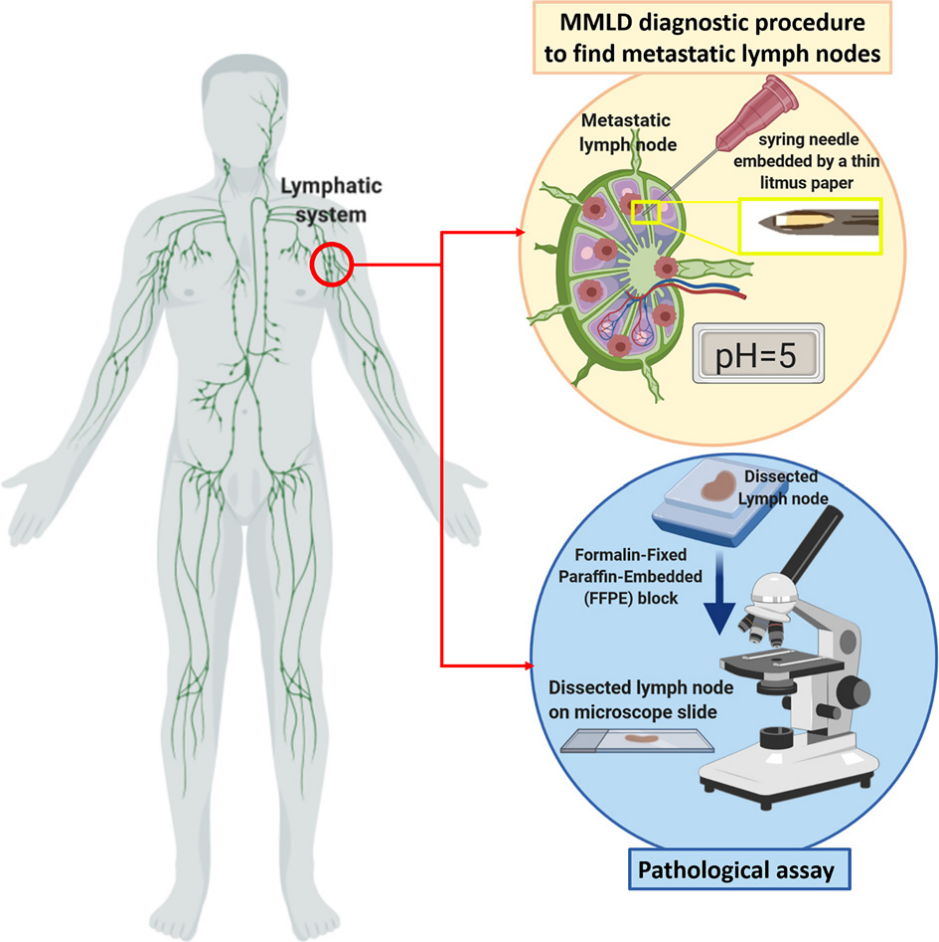
The schematic of the MMLD and test procedure for detecting cancer cells in the suspicious lymph nodes During surgery, the lymph node is sent for the frozen pathology as an only common method for the detection of metastatic cancer cells in the sentinel/auxiliary lymph nodes.

Hence by real-time and precise pH sensing of LNs, we might achieve a calibration pattern to distinguish normal from metastatic LNs. To achieve this calibration, PBS (100 µl) was injected to SLNs immediately after dissection and MMLD was entered to the LNs. Then, the pH was read due to the color of litmus paper. After pathological evaluation of the LNs, the pH and diagnosis of each LNs were recorded in Supplementary Table S1. A schematic diagram of MMLD made to detect the acidity or basicity of a lymph node fluid is shown in Figure 2. All of these sensors are sterilized after being manufactured under the plasma sterile protocol (Standard Number: ISO / NP 22441).

**Figure 3:**
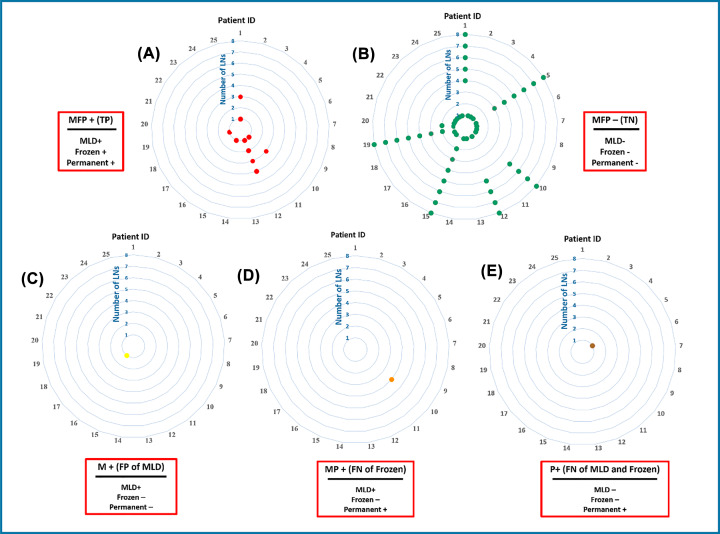
MMLD scoring on lymph node samples versus pathological diagnoses (Frozen and permanent H&E) of 25 breast cancer patients; permanent H&E is the gold standard (**A**) The number of patient’s ID which all three MMLD/Frozen/permanent declared positive (MFP+). (**B**) Number of patient’s ID that which all three MMLD/Frozen/permanent were negative (MFP−). (**C**) Patient ID 16 (sample ID 48) which MMLD was positive but both frozen and permanent H&E declared negative (False positive of MMLD). (**D**) Patient ID 10 (sample ID 25) which MMLD and permanent were positive but frozen declare negative (false negative of frozen). (**E**) Patients ID 5 (sample ID 12) which both MMLD and Frozen declared negative but permanent rejected their diagnosis (false negative of MMLD and frozen). Internal circles in each diagram indicate the number of tested LNs for one patient. Numbers 1 to 8 refer to sentinel lymph node 1, sentinel lymph node 2 and auxiliary lymph nodes 1 to 6 in circle lines for each patient.

This method has been tested on 65 lymph nodes immediately after dissection (through standard guidelines) from 25 breast cancer patients. Some of them were normal and some others were metastatic due to pathological evaluations (Supplementary Table S1). Changes in the pH value of the LNs from more than 7.0 in healthy LNs to less than 6.0 in cancerous ones indicate well differentiation ability of the lymphatic fluid (LF) pH as metastasis indicator. The results of the MMLD sensor were compared by frozen section diagnostics based on permanent H&E of the lymph nodes as the gold standard. Among 65 samples, MMLD had 1 false-positive and 1 false-negative. Figure 3 showed the comparative circle diagrams of the MMLD, Frozen, and Permanent pathology responses on 65 healthy and cancerous lymph nodes.

MMLD showed approximately 90% selectivity and 92% sensitivity detecting cancer cells metastasized to lymph nodes while this value in conventional frozen pathology was both 83% (Table 1; Supplementary Tables S2 and S3).

**Table 1:** Comparative diagnostic results of the MMLD and frozen pathology based on the permanent pathology as a gold standard for 65 LNs samples from 25 patients

*Diagnosis results of the LNs (65 samples from 25 breast cancer patients)*	*MMLD*	*Frozen Pathology*
*TP*	11	10
*TN*	52	53
*FP*	1	0
*FN*	1	2
*Sensitivity*	92%	83%
*Specificity*	98%	100%
*Selectivity*	90%	83%

Abbreviations: FN, false negative; FP, false positive; TN, true negative; TP, true positive.

**Figure 4:**
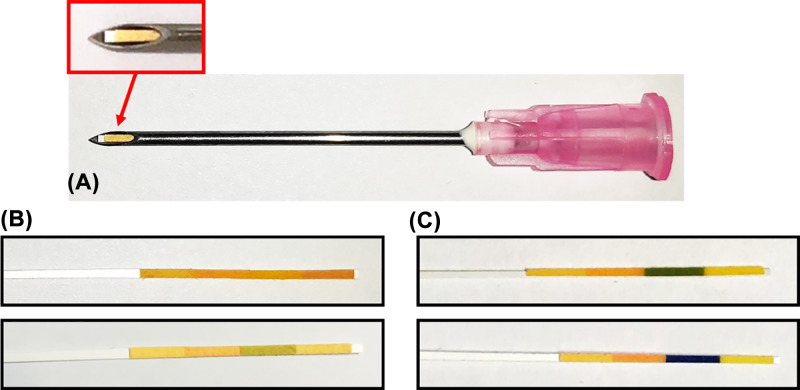
MMLD responses to the pH of the SLN secreted fluid (**A**) MMLD for the detection of acidic or basic in lymph node secretion solution. MMLD response to pH of the lymph secreted solution. The color of pH-indicator strips changes from lymph node secretion, (**B**) Cancer (less than pH 6.0), and (**C**) healthy (pH = 7.0–10.0).

The pH of the SLN fluid secreted in the cancerous lymph was less than 6.0 and for healthy ones were 7.0–10.0, respectively (Figure 4).

**Figure 5:**
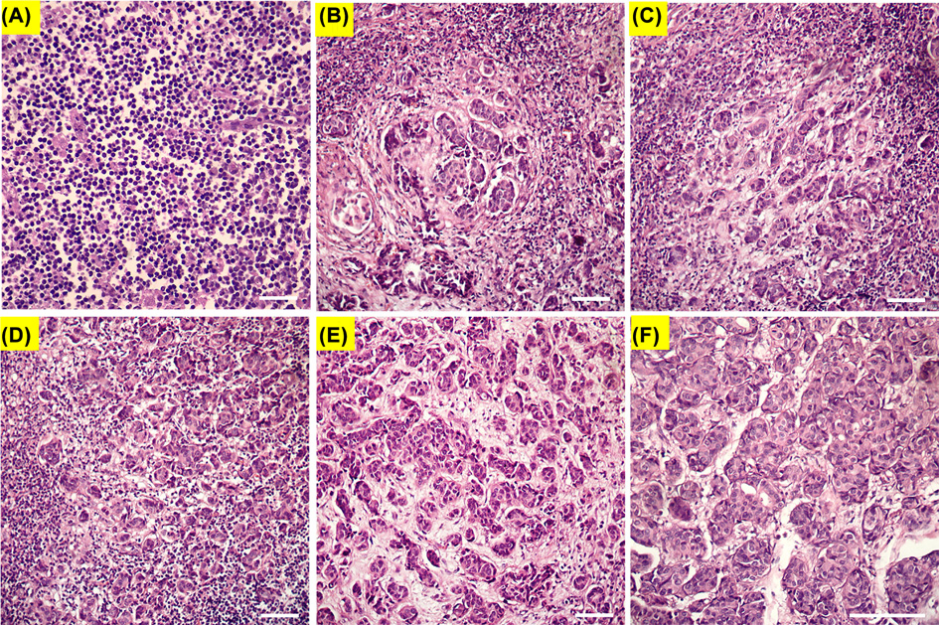
The H&E image of the cancerous lymph node with different percent of cancer cells that detected by MMLD (**A**) Normal lymph node (pH = 10.0; patient ID 4 and sample ID 11), lymph nodes with (**B**) 20% (pH = 6.0; patient ID 10 and sample ID 25), (**C**) 50% (pH = 5.5; patient ID 10 and sample ID 24), (**D**) 85% (pH = 5.0; patient ID 12 and sample ID 32), (**E**) 90% (pH = 4.0; patient ID 12 and sample ID 31), (**F**) more than 95% (pH = 4.0; patient ID 1 and sample ID 1) of cancer cells. Each bar is equal to 100 μm.

The H&E images of the normal and cancerous lymph nodes with different percentage of involved cancer cells were shown in Figure 5. One case of involved lymph nodes detected by MMLD but missed in the frozen, based on permanent pathology gold standard, is shown in Figure 5B (patient ID 10).

## Conclusion

In summary, the MMLD was designed to detect metastatic cancer cells in the lymph node during surgery. This tool can also be very effective in the surgical process to prevent the removal of healthy lymph nodes. Also, it can help to make an accurate and real-time diagnosis in the operating room with no need for unnecessary secondary surgery and can reduce recurrences. Another application of this system would be in radiological process to help interventional radiologist for better identification of involved LNs.

## Supplementary Material

Supplementary Tables S1-S3

## Abbreviations

ALN: auxiliary lymph node
FAO: fatty acid oxidation
LF: lymphatic fluid
LN: lymph node
MMLD: metabolism based metastatic lymph diagnoser
PBS: phosphate buffer saline
SLN: sentinel lymph node
YAP: Yes-Associated Protein
