# Supplementary material for: Real-time diagnosis of sentinel lymph nodes involved to breast cancer based on pH sensing through lipid synthesis of those cells
Source: Biosci Rep. 2020 Jun 8;40(6):BSR20200970. doi: 10.1042/BSR20200970 (PMC7280473; doi:10.1042/BSR20200970)
Supplement: Supplementary Tables S1-S3 [file BSR-2020-0970_supp.pdf]

Supplementary Table 1. Direct comparisons between MMLD responses and pathological assay MMLD scoring on lymph node samples vs. pathological diagnoses of 25 breast cancer patients. Positive samples indicated with Red (+), negative samples indicated with green (-).During this test two samples assumed as MMLD false (one false positive and false negative: patient's ID 16 (sample ID 48) and 5 (sample ID 12)). This test individually repeated by three needles and if even one of the needles showed acidic pH with pH lower than 6.0 (as experimentally was calibrated), then the LN would be declared as involved LNs.

Patient ID #	Patient samples #	Type of lymph node	MMLDdiagnosis (pH value)	Frozen pathology diagnosis	Permanent pathology diagnosis	Sensor response compare to Permanent pathology (gold standard)
**1**	1	Sentinel	4.0	+	+	TP
**1**	2	Auxiliary 1	5.0	+	+	TP
**1**	3	Auxiliary 2	7.0	-	-	TN
**1**	4	Auxiliary 3	10.0	-	-	TN
**1**	5	Auxiliary 4	7.0	-	-	TN
**1**	6	Auxiliary 5	8.0	-	-	TN
**1**	7	Auxiliary 6	8.0	-	-	TN
**2**	8	Sentinel 1	8.0	-	-	TN
**2**	9	Sentinel 2	7.0	-	-	TN
**3**	10	Sentinel	7.0	-	-	TN
**4**	11	Sentinel	10.0	-	-	TN
**5**	12	Sentinel	7.0	-	+	FN
**5**	13	Auxiliary 1	7.0	-	-	TN
**5**	14	Auxiliary 2	7.0	-	-	TN
**5**	15	Auxiliary 3	7.0	-	-	TN
**5**	16	Auxiliary 4	9.0	-	-	TN
**5**	17	Auxiliary 5	8.0	-	-	TN
**5**	18	Auxiliary 6	8.0	-	-	TN
**6**	19	Sentinel	10.0	-	-	TN
**7**	20	Sentinel	10.0	-	-	TN
**8**	21	Sentinel	7.0	-	-	TN
**9**	22	Sentinel	7.0	-	-	TN
**10**	23	Sentinel	4.0	+	+	TP
**10**	24	Auxiliary 1	5.5	+	+	TP
**10**	25	Auxiliary 2	6.0	-	+	TP
**10**	26	Auxiliary 3	7.0	-	-	TN
**10**	27	Auxiliary 4	7.0	-	-	TN

**10**	28	Auxiliary 5	8.0	-	-	TN
**10**	29	Auxiliary 6	8.0	-	-	TN
**11**	30	Sentinel	7.0	-	-	TN
**12**	31	Sentinel 1	4.0	+	+	TP
**12**	32	Sentinel 2	5.0	+	+	TP
**12**	33	Auxiliary 1	6.0	+	+	TP
**12**	34	Auxiliary 2	6.0	+	+	TP
**12**	35	Auxiliary 3	10.0	-	-	TN
**12**	36	Auxiliary 4	8.0	-	-	TN
**12**	37	Auxiliary 5	8.0	-	-	TN
**12**	38	Auxiliary 6	10.0	-	-	TN
**13**	39	Sentinel	10.0	-	-	TN
**14**	40	Sentinel	7.0	-	-	TN
**15**	41	Sentinel	6.0	+	+	TP
**15**	42	Auxiliary 1	8.0	-	-	TN
**15**	43	Auxiliary 2	9.0	-	-	TN
**15**	44	Auxiliary 3	9.0	-	-	TN
**15**	45	Auxiliary 4	7.0	-	-	TN
**15**	46	Auxiliary 5	7.0	-	-	TN
**15**	47	Auxiliary 6	7.0	-	-	TN
**16**	48	Sentinel	6.0	-	-	FP
**17**	49	Sentinel	8.0	-	-	TN
**18**	50	Sentinel	8.0	-	-	TN
**19**	51	Sentinel	5.0	+	+	TP
**19**	52	Auxiliary 1	7.0	-	-	TN
**19**	53	Auxiliary 2	7.0	-	-	TN
**19**	54	Auxiliary 3	10.0	-	-	TN
**19**	55	Auxiliary 4	10.0	-	-	TN
**19**	56	Auxiliary 5	7.0	-	-	TN
**19**	57	Auxiliary 6	8.0	-	-	TN
**20**	58	Sentinel 1	7.0	-	-	TN
**20**	59	Sentinel 2	7.0	-	-	TN
**21**	60	Sentinel	9.0	-	-	TN
**22**	61	Sentinel	8.0	-	-	TN
**23**	62	Sentinel	7.0	-	-	TN
**24**	63	Sentinel 1	7.0	-	-	TN
**24**	64	Sentinel 2	10.0	-	-	TN
**25**	65	Sentinel	8.0	-	-	TN

Supplementary Table 2. Confusion matrix for MMLD predicted results based on pathological assays (as gold standard) for 65LNs samples from 25 patients(TP: True Positive, FP: False Positive, TN: True Negative, and FN: False Negative).

		Actual value (as confirmed by permanent pathology (gold standard))		
	N=65	**positive**	**negative**	
Predicted value (predicted by MMLD)	**positive**	TP=11	FP=1	12
	**negative**	FN=1	TN=52	53
		12	53	

Misclassification rate[1]= 0.03

Sensitivity[2] = 9 2%

Specificity[3] =98%

Selectivity[4]= 90%

---

[1]Misclassification rate = $\frac{\mathbf{FP + FN}}{\mathbf{total}}$

[2]True positive rate (sensitivity) = $\frac{TP}{actualpositive}$

[3]True negative rate (specificity) = $\frac{TN}{actualnegative}$

[4]Selectivity= true positive rate × true negative rate

Supplementary Table 3. Confusion matrix for Frozen predicted results based on pathological assays (as gold standard) for 65LNs samples from 25 patients. (TP: True Positive, FP: False Positive, TN: True Negative, and FN: False Negative).

		Actual value (as confirmed by permanent pathology (gold standard))		
	**N=65**	**positive**	**negative**	
**Predicted value (predicted by Frozen)**	**positive**	TP=10	FP=0	10
	**negative**	FN=2	TN=53	55
		12	53	

Misclassification rate=0.03

Sensitivity =83%

Specificity =100%

Selectivity=83%
